# Once upon Multivariate Analyses: When They Tell Several Stories about Biological Evolution

**DOI:** 10.1371/journal.pone.0132801

**Published:** 2015-07-20

**Authors:** Sabrina Renaud, Anne-Béatrice Dufour, Emilie A. Hardouin, Ronan Ledevin, Jean-Christophe Auffray

**Affiliations:** 1 Laboratoire de Biométrie et Biologie Evolutive, UMR5558, CNRS, University Lyon 1, 69622 Villeurbanne, France; 2 Max Planck Institute of Evolutionary Biology, August-Thienemann-Str. 2, Plön, Germany; 3 Faculty of Science and Technology, Bournemouth University, Christchurch House, Talbot Campus, Poole, Dorset, United Kingdom; 4 Institut des Sciences de l’Evolution de Montpellier, UMR 5554, CNRS, University Montpellier 2, Montpellier, France; Monash University, AUSTRALIA

## Abstract

Geometric morphometrics aims to characterize of the geometry of complex traits. It is therefore by essence multivariate. The most popular methods to investigate patterns of differentiation in this context are (1) the Principal Component Analysis (PCA), which is an eigenvalue decomposition of the total variance-covariance matrix among all specimens; (2) the Canonical Variate Analysis (CVA, a.k.a. linear discriminant analysis (LDA) for more than two groups), which aims at separating the groups by maximizing the between-group to within-group variance ratio; (3) the between-group PCA (bgPCA) which investigates patterns of between-group variation, without standardizing by the within-group variance. Standardizing within-group variance, as performed in the CVA, distorts the relationships among groups, an effect that is particularly strong if the variance is similarly oriented in a comparable way in all groups. Such shared direction of main morphological variance may occur and have a biological meaning, for instance corresponding to the most frequent standing genetic variation in a population. Here we undertake a case study of the evolution of house mouse molar shape across various islands, based on the real dataset and simulations. We investigated how patterns of main variance influence the depiction of among-group differentiation according to the interpretation of the PCA, bgPCA and CVA. Without arguing about a method performing ‘better’ than another, it rather emerges that working on the total or between-group variance (PCA and bgPCA) will tend to put the focus on the role of direction of main variance as line of least resistance to evolution. Standardizing by the within-group variance (CVA), by dampening the expression of this line of least resistance, has the potential to reveal other relevant patterns of differentiation that may otherwise be blurred.

## Introduction

In the last few decades, geometric morphometrics [1, 2] has been established as a powerful tool to characterize shape variation of complex morphological structures, in contexts as diverse as phylogenetic diversification [3, 4], developmental biology [5, 6] and response to environment [7]. As geometric morphometrics aims to characterize the geometry of complex traits, it is inherently multivariate. The most commonly-used multivariate analysis in geometric morphometrics is probably the Principal Component Analysis (PCA) [8]. It is an eigenvalue decomposition of the total variance-covariance matrix among all specimens. It is especially used when looking at patterns of morphological variance (e.g. [9–11]). The Canonical Variate Analysis (CVA) has emerged as a popular and powerful way to investigate patterns of differentiation among groups (e.g. [12–14]). It corresponds to a linear discriminant analysis (LDA) for more than two groups [15, 16]. CVA aims at separating the groups by looking for linear combinations of variables that maximize the between-group to within-group variance ratio [17]. Recently, the between-group PCA (bgPCA) [18]has emerged as a potential alternative to CVA to investigate patterns of among group differentiation, without standardizing for the within-group variance [19, 20].

Building a biological interpretation of morphometric variation profoundly relies on the image that multivariate analyses provide of the pattern of differentiation. Standardizing within-group variance, as performed in the CVA, distorts the relationships among groups [21, 22]. This effect will be particularly strong if the variance is similarly oriented (anisotropic) in all groups, because in this case standardizing by within-group variance will correspond to dampen differences among groups along this particular direction. A preferred direction of variance parallel among groups is not only a theoretical issue: Biological mechanisms can lead to such patterns. The direction of main morphological variance is related to the most frequent standing genetic variation in a population [23, 24]. By providing frequent variants to the screening of selection, these directions of main variance have been proposed to constitute lines of least resistance to evolution [9, 25].

**Fig 1:**
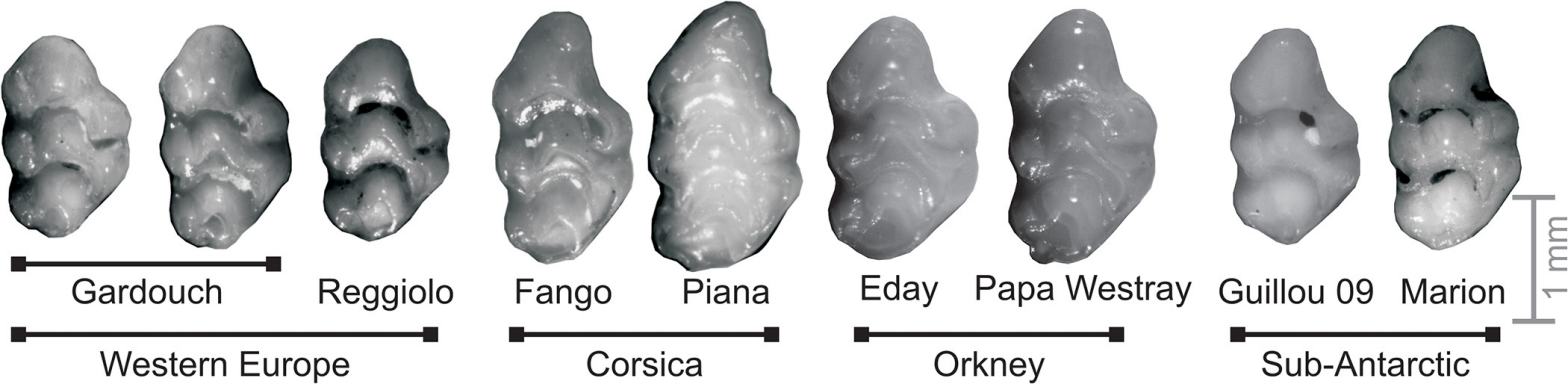
Mouse teeth exemplifying the morphological variation within and between populations. From left to right: Western European mainland populations (Gardouch, South France; Reggiolo, Northern Italy); insular populations: Corsica (Fango, Corsica mainland; Piana islet); Orkney (Eday and Papa Westray islands); sub-antarctic islands (Guillou, island part of the Kerguelen archipelago and Marion, part of Prince Edward islands). Anterior part to the top, lingual side to the right.

In this context, one can wonder how much a multivariate method will be sensitive to such biologically-relevant patterns of main variance, and how much this will influence the depiction of among-group differentiation offered to interpretation. We address here these issues using a case study: the evolution of house mouse molar shape across various islands. The mouse molar (Fig 1) has been shown to display directions of main variance shared among populations [26] that seems to constitute preferential directions of among-group differentiation [27]. In this case study, we (1) compared the pattern of among-group differentiation provided by three popular multivariate analyses in geometric morphometrics: PCA, bgPCA and CVA; (2) tested on real and simulated data sets how far these patterns were influenced by directions of main variance shared among groups; and (3) questioned the biological relevance of interpretations based on the among-group patterns provided by the various methods. Without arguing about one method performing ‘better’ than another, it rather emerges that working on the total or between-group variance (PCA and bgPCA) will tend to put the focus on the role of direction of main variance as line of least resistance to evolution. Standardizing by the within-group variance (CVA), by dampening the expression of this line of least resistance, has the potential to evidence other relevant patterns of differentiation that may otherwise be blurred.

### Case study: molar shape divergence of house mice on islands

**Fig 2:**
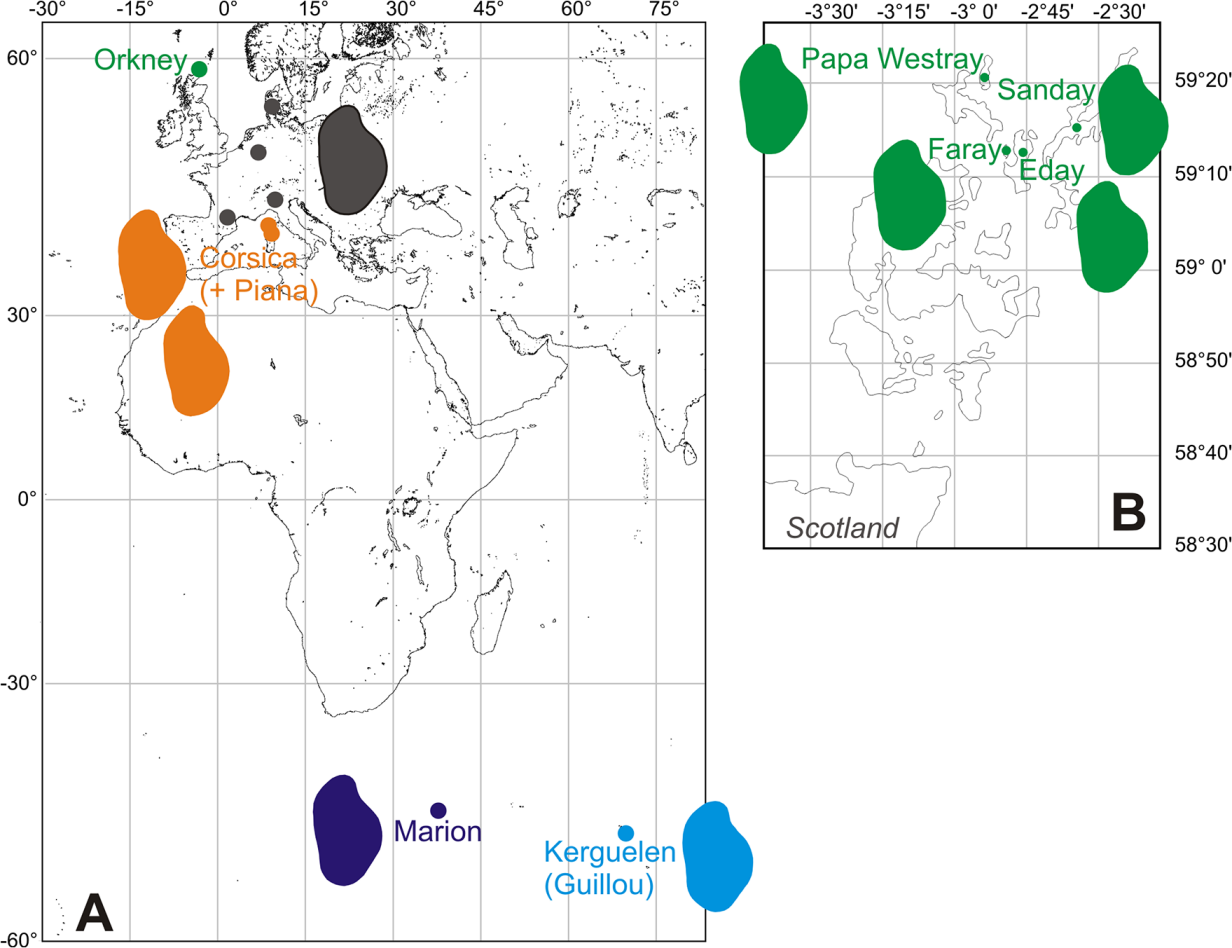
Location of the studied populations. A. General map, with dots showing sampling localities: Western European mainland areas (in grey) and archipelagos (Orkney, in green; Corsica, in orange; Marion, in dark blue; Kerguelen, light blue). B. Close-up on Orkney archipelago, with the four islands sampled. Colored shapes correspond to reconstruction of the mean outline per population or area is represented (for Guillou, year 2009).

The house mouse (*Mus musculus domesticus*) is a highly successful colonizer because it accompanied human travels since archaeological times [28, 29]. This has led to a complex phylogeographic pattern across worldwide populations of mice (e.g. [30–32]). This genetic structure is mirrored by differences in molar shape among continental stocks [33, 34]. The house mouse also successfully colonized many islands [29]. Insular conditions are known to promote fast and pronounced morphological differentiation because of peculiar ecological conditions triggering adaptations, as well as due to random processes such as founder effect and subsequent drift in small and isolated populations [35, 36]. As in other rodents, house mice are sensitive to such factors and cases of marked morphological differentiation have been reported for several islands [29, 37–39]. Our data set included continental and insular populations from various environmental contexts (Fig 2). Phylogenetic relationships were assessed based on data from the literature, and compared to the pattern of morphological differentiation, as provided by the different multivariate methods (PCA, bgPCA, and CVA) applied to the same morphometric dataset.

## Material

A total of 432 first upper molars were considered in the analyses (Table 1; Fig 2). All mice were sub-adults and adults, the criteria being the eruption of the third molars that occurs at weaning. Sexual dimorphism has not been documented so far in molar shape of wild mouse populations [37, 40]. All animals were therefore pooled in subsequent analyses.

**Table 1:** Samples used in this study. Area and country/island are indicated, locality of trapping, labels used in the figures, and number of first upper molars (UM1) measured.

Area	Sub-Area	Locality	Labels	UM1
Continent	France	Gardouch	FR-GARD	68
		Montpellier	FR-MONTP	13
	Italy	Lombardy	IT-LOMB	15
		Reggiolo	IT-REG	7
		San Bernardino	IT-SBER	18
	Germany	Cologne-Bonn	GER-CB	14
	Denmark	Egtved	DK-EGTV	14
Corsica	Corsica	Fango Valley	CO-NW	53
	Piana	Piana	PIANA	6
Orkney		Eday	O-EDAY	18
		Faray	O-FARAY	12
		Papa Westray	O-PW	10
		Sanday	O-SANDAY	8
Sub-Antarctic	Kerguelen	Guillou 1993	G1993	22
		Guillou 2001	G2001	20
		Guillou 2008	G2008	20
		Guillou 2009	G2009	22
	Marion	Marion	MARION	92

Seven populations from Western Europe were used to represent the continental variation in house mice, including samples from France (Montpellier, Gardouch), Italy (Reggiolo, San Bernardino and farms from the surroundings [Lombardy]), Germany (Cologne and Bonn surroundings) and Denmark (Egtved).

Insular populations were used to examine various archipelagos and environmental contexts. Corsica is a large Mediterranean island. It was sampled by mice from Fango valley and from the islet of Piana, a few kilometers off Corsica [26]. The Orkney archipelago is located in North Atlantic, 16 km off the coast of Scotland. Mice from four islands were included in the present study (Sanday, Faray, Eday, Papa Westray). Finally, sub-antarctic conditions were documented by mice from Marion Island, at latitude ~46°S some 1770 km off South Africa, and by populations from the small Guillou Island, in the Kerguelen archipelago. It is located in the Indian Ocean about 4000 km away from African and Australian coasts, at latitude ~50°S. Samples from Guillou were trapped in four years spanning over more than 15 years (1993, 2001, 2008 and 2009) [37].

Mice from Montpellier, Corsica, Marion, and Orkney come from the collection of the Institut des Sciences de l’Evolution, Montpellier, France. Mice from Gardouch have been trapped by Jean-Pierre Quéré and are deposited at the Centre de Biologie et Gestion des Populations, France. Mice from Guillou have been trapped by the team of Jean-Louis Chapuis and Benoit Pisanu (Museum National d’Histoire Naturelle, Paris, France) and those from Cologne-Bonn by team of the Max Planck Institute for Evolutionary Biology, Plön, Germany. The corresponding skulls have been prepared and are presently stored at the Laboratoire de Biométrie et Biologie Evolutive, Lyon, France. All mice were sacrificed after trapping according to the 2010/63/UE directive. Authorizations: permit to Jean-Christophe Auffray C34-130 [préfecture de l’Hérault]; permit to Jean-Pierre Quéré 34–107 [préfecture de l’Hérault]; permit to Jean-Louis Chapuis 68–013 [Comité Cuvier d’Ethique]; permit for mice from Cologne Bonn: V312-72241.123–34 and approval by the ethics commission of the Ministerium für Landwirtschaft, Umwelt und ländliche Räume, Kiel (Germany) [41]. According to the French legislation, sacrifice of wild animals for the purpose of taking samples, when performed according to authorized protocols, is not considered as an experiment (Journal Officiel de la République Française, Décret n° 2013–118 du 1er février 2013, Section 6, Sous-section 1). As such, it is not submitted to the agreement of ethical committees.

## Methods

### Phylogenetic analyses

A total of 424 mitochondrial (mt) control region sequences of 834 bp were extracted from GenBank:

- France: 151 [30, 42, 43]; Jones et al unpubl.;
- Germany: 71 [30, 42, 43]; Jones et al unpubl.;
- Italy: 60 [31, 44, 45] and Jones et al unpubl.;
- Denmark: 23 [45];
- Orkney: 21[32, 44, 45]
- Corsica: 1 [46]
- Marion Island: 18 [47]
- and Guillou Island [47].

These sequences were extracted and aligned using BioEdit [48] and MEGA [49]. The HKY+I substitution model was selected using jmodeltest-2.1.4 [50]. Genetic distances were calculated using the software Mr Bayes [51] with the following conditions, 25% burn-in and 5 000 000 generations using *Mus musculus musculus* DQ266060.1 as an outgroup. Based on these distances, the final tree was visualized using FigTree 1.3.1 [52].

### Outline analysis

The molar shape was approximated by the 2D outline of the tooth seen from the occlusal surface, the focus being made towards the base of the crown, which is only affected by heavy wear [53]. Each outline was defined by a set of 64 points, the starting point being tentatively positioned at the anteriormost part of the tooth. This set of points was first analyzed using an Elliptic Fourier transform [54]. This method describes the outline as its x- and y-variations, as a function of the cumulative length along the outline, decomposed into a sum of trigonometric functions of decreasing wavelength (harmonics) by a Fourier approach. Each harmonic is weighted by four Fourier coefficients (FCs), two for x- and two for y-variations. This method allows the alignment of the outline along the first axis of the ellipse best fitting the outline, and the adjustment of the starting point at the intersection of the outline with this first axis, corresponding to the major elongation of the object. A drawback is a high number of variables required to describe the outline (four FCs by harmonics). This method was used to reconstruct an outline with the starting point adjusted along the first axis of the major ellipse. This outline was then analyzed using a Fourier method decomposing the distance of each point to the center of gravity of the outline as a function of the distance along the outline. Each harmonic is weighted by two Fourier Coefficients (FCs) using this method, reducing the dataset required to describe the outline. The zero harmonic, proportional to the outline size, was used as size estimator and to standardize all other FCs so they represent shape variables only. The higher the rank of the subsequent harmonics, the more details they represent on the outline. The shape of a mouse molar is adequately described by the first seven harmonics, i.e. by 14 variables [26] (dataset: S1 Table).

### Multivariate analyses of shape variation

A Principal Component Analysis (PCA) on the total variance-covariance (VCV) matrix **T** was performed on the set of the 14 FCs describing molar tooth outlines to study total shape variation among specimens. This method is an eigenanalysis of **T** providing axes maximizing the variance among all specimens.

Variance between groups (here, localities) was first assessed using a between-group PCA (bgPCA). The total VCV matrix **T** is decomposed in two components: the between-group matrix **B** and the within group matrix **W**. **B** corresponds to the VCV between group means weighted by the sample size of each group. **W** is equal to **T–B**. The bgPCA corresponds to the eigenanalysis of **B**.

An alternative approach to study differences among groups is to use a canonical variate analysis (CVA), an extension of the linear discriminant (LDA) analysis to more than two groups [15, 16]. This analysis provides axes maximizing the between-group to within-group variance ratio. It corresponds to an eigenanalysis of **BW** ^**-1**^. Note that CVA presents computational problems when number of variables is larger than number of cases. It may require a reduction of dimensionality of the data [21]. This is not an issue here because using Fourier methods allows for a thresholding of the number of variables before statistical analyses.

Multivariate analyses were performed under R [55] using the package ade4 [56].

### Characterization of the direction of main variance in well-sampled groups

The standardization by the intragroup variance **W** should impact CVA more if groups displayed marked directions of main variance parallel in the different groups.

Such direction of main variance for a group i corresponds to the first eigenvector of a PCA on the total VCV matrix for the group i, i.e. **T**_**i**_. This phenotypic VCV matrix is also called **P** (**P**_**i**_ for group i). Its first eigenvector is called Pmax for direction of maximum phenotypic variance [23]. Its estimates requires a large sample size, i.e. ideally more than 30 specimens for the concerned group [27, 57]. It was therefore assessed in the three well-sampled groups of Gardouch (mainland Western Europe), Corsica and Marion Island.

The concordance of the estimates of Pmax in these three groups was assessed by a bootstrapping procedure. Each group was bootstrapped 100 times, corresponding Pmax _bootsrap_ were estimated and compared to Pmax estimated on the original samples. The distribution of the correlation R between Pmax _original_ and the 100 Pmax _bootsrap_ provided a ‘confidence interval’ to which the correlation between Pmax of two different groups was compared. If R _observed between group_ was within the 95% distribution of R _original / bootstrap_, it was considered that the Pmax in two concerned groups could not be considered as different.

### Comparison between patterns of differentiation provided by the different multivariate methods

The representations of differentiation provided by the different multivariate methods applied to the same dataset (molar shape) were compared as follow. The scores of the group means on axes of a given analysis provide a configuration that can be compared to the configuration of group means as provided by another method. Such configurations can be compared using a Procrustes superimposition (Protest) [58]. This provides a measure of Procrustes distance between the two configurations (D) and a coefficient of correlation R. The probability that the configurations are more related than random is assessed using permutations.

As a way to assess how some multivariate methods applied to morphometric datasets may or may not favor an evolutionary hypothesis, the patterns of shape differences were compared to the genetic differentiation. In order to do so, the matrix of genetic distance was converted into a set of multivariate axes by using a Principal Coordinate Analysis (PCoA) [59]. Based on the genetic data available in the literature, groups as close as possible as the ones used for morphometric analyses were identified. Their means on the PCoA axes were computed, providing a configuration that was compared to the configurations provided using the morphometric analyses using a Protest. *pcoa* and *protest* are R functions from the package ‘vegan’ [60].

### Impact of oriented within group variance on the output of multivariate analyses: simulations

In order to assess the effect of an ‘anisotropic’ within-group variance (i.e. oriented along a preferential direction shared among groups) on the output of PCA, bgPCA and CVA, simulations were performed. 999 datasets were built from the same seven populations and the same individuals per population observed in the real dataset. For each simulated dataset, the seven populations kept their observed means. However, the variance of each variable within each population was modified to follow a same variance. This was done for each population separately by randomizing the simulated values from a multinormal distribution with the following parameters: the 14 observed means characteristic of the given population, and as variance the average of the 7 geometric means of the 14 variances per population. This was repeated independently for each of the 7 populations. PCA, bgPCA and CVA were performed on these simulated datasets. Three pairwise Protests were computed to compare the among-group configuration obtained on four axes of each analysis (PCA vs. bgPCA, PCA vs. CVA, bgPCA vs. CVA). For each comparison, the 999 Protest distances from the simulated datasets generated a simulated distribution function. The observed distances between analyses from the real dataset were compared to this simulated function. The script in R is provided as supplementary file (S1 Script).

## Results

### Phylogenetics: Independent colonization of the different archipelago

**Fig 3:**
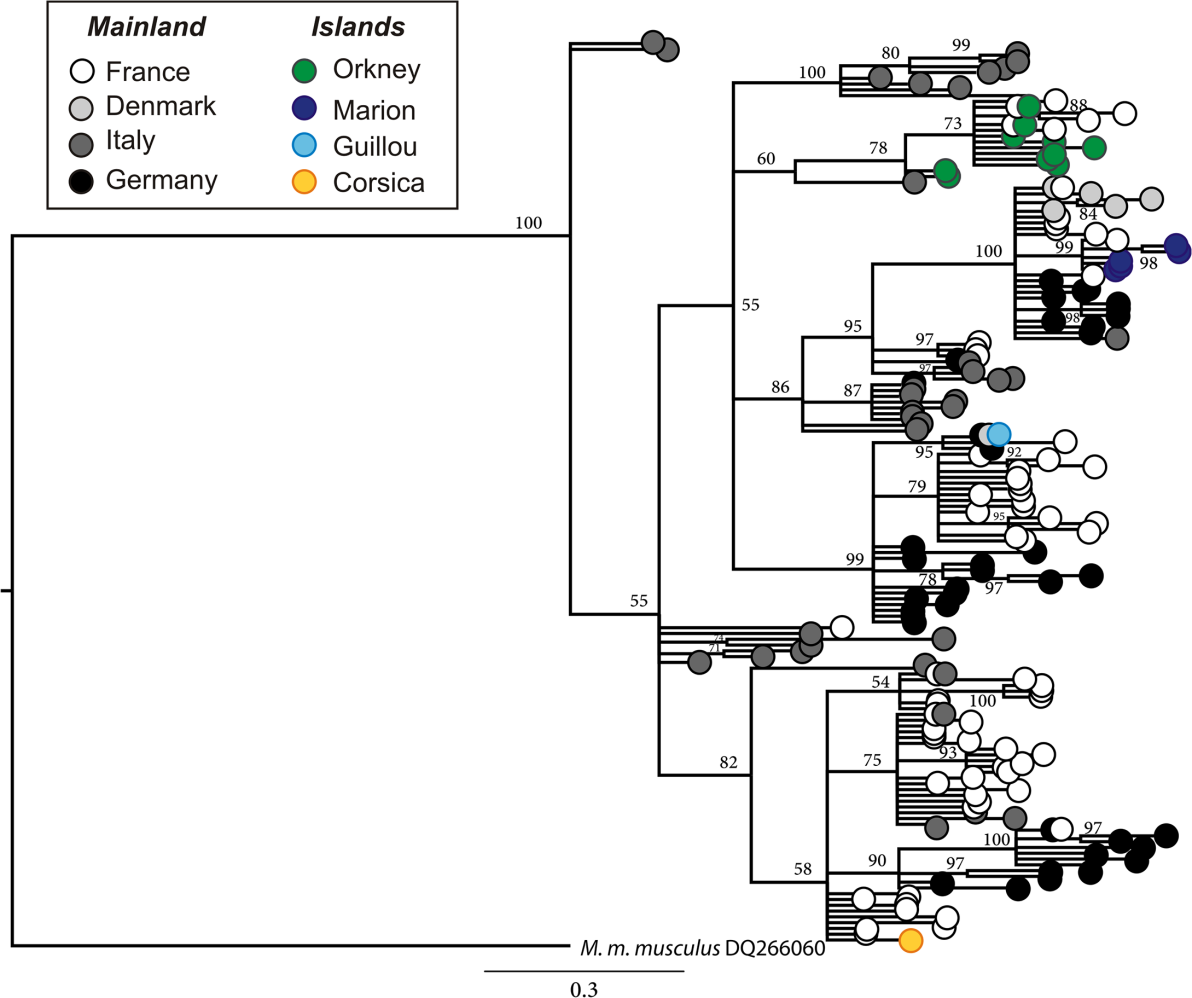
Phylogenetic tree for *M*. *m*. *domesticus* mt DNA control region. The tree includes mice from France, Denmark, Italy, Germany, Marion Island, Guillou Island, Corsica and Orkney (based on data available in GenBank). The tree was drawn after Bayesian analysis and shows the different origins of the island mouse populations.

A total of 424 mt DNA control region sequences of 834 bp were obtained from GenBank. The populations from the four archipelagos are found on different branches of the phylogenetic tree, suggesting that Orkney, Guillou Island, Marion Island and Corsica were colonized by different founder populations from Europe (Fig 3).

### Patterns of molar shape differentiation

#### PCA

**Fig 4:**
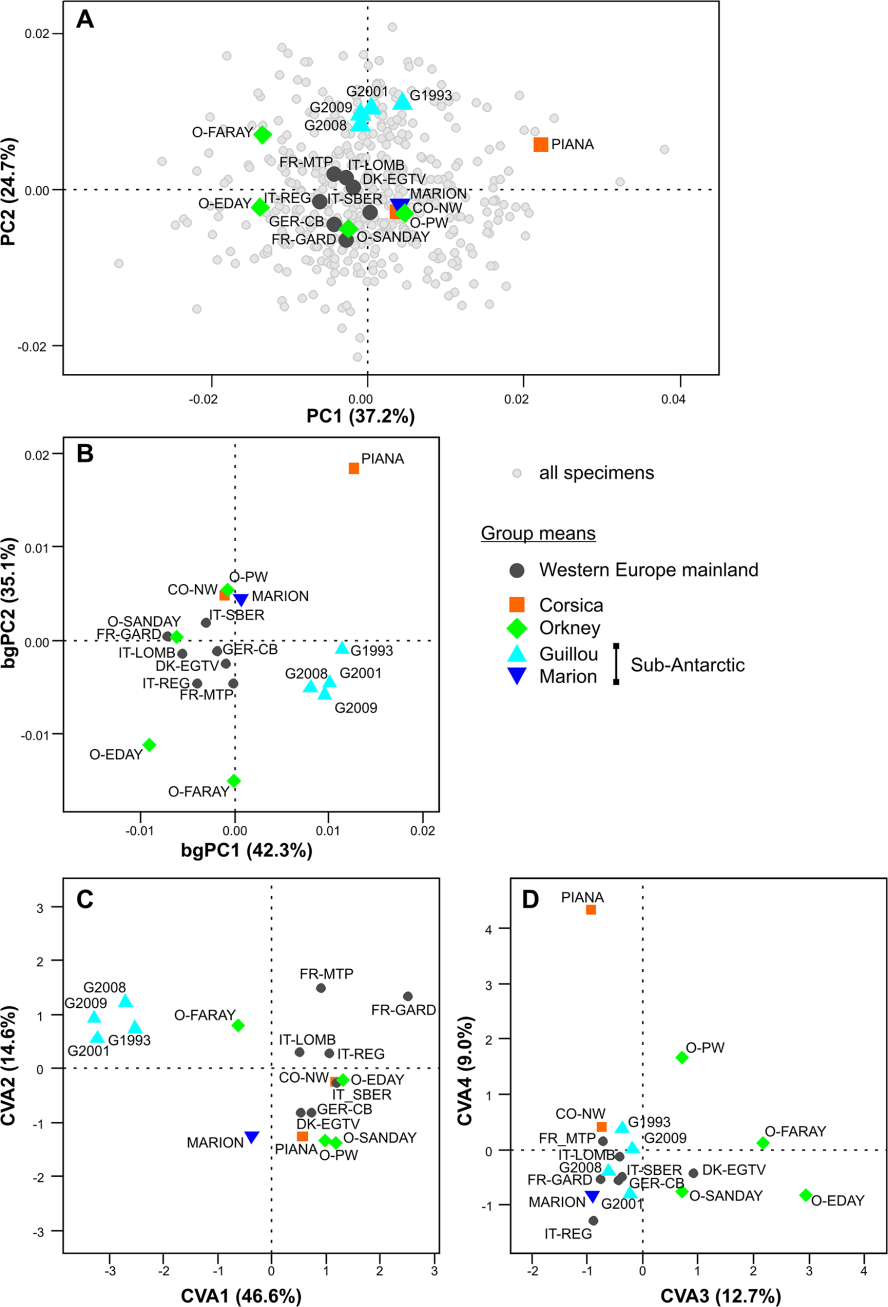
Differentiation in molar shape among populations, according to three different multivariate analyses. A. Principal Component Analysis (PCA); note that this analysis is performed on all specimens (grey dots). Group means are highlighted (large colored symbols). B. Between-group PCA. C and D. Canonical Variate Analysis (CVA), in C first vs. second axes, in D third vs. fourth axes.

All continental localities from Western Europe cluster together. Insular groups scatter around this continental cluster (Fig 4A). The first axis (37.2% of the total variance) is characterized by the strong divergence of Piana, and to a lesser extent of Corsica, Papa Westray (Orkney) on one side, and from Faray and Eday (Orkney) on the other side. The second axis (24.7% of variance) mostly isolates samples from Guillou. No clear pattern emerges on axes 3 and 4 (PC3 = 10.5%, PC4 = 7.6%; data not shown).

#### bgPCA

The pattern of differentiation (Fig 4B) is close to the one observed based on a PCA on the total variance. The main pattern of differentiation, opposing Piana to Faray and Eday, is oblique to the first axis (42.3% of between-group variance) and second axis (35.1%). No clear pattern emerges on axes 3 and 4 (bgPC3 = 8.0%, bgPC4 = 6.9%; data not shown).

#### CVA

The main pattern of differentiation isolates the samples of Guillou from the cluster of mainland groups along the first axis (46.6% of among-group variance). Along the second axis (14.6%), various insular groups (Marion, Piana, Sanday and Papa Westray) tend to segregate from the mainland cluster. The third axis (12.7%) differentiates Faray, Eday and Papa Westray and to a lesser degree Sanday (all from Orkney archipelago). Piana strongly diverges along the fourth axis (9.0%).

### Relationships between patterns of differentiation

#### Morphometric patterns of differentiation

The configuration between the 18 group means provided by the different analyses were compared using a Protest (999 permutations). In order to compare these configurations, the same numbers of axes have to be considered for all analyses, the only first four axes were retained (results based on the first two axes provided similar results; data not shown). Since the PCA, bgPCA and CVA were based on the same morphometric data set, the resulting patterns were of course correlated. The PCA and bgPCA provided almost similar configurations (R^2^ = 0.960; Procrustes Distance D = 0.078; P_999 permutations_ = 0.001). The pattern of the CVA provided a pattern three times more distant (CVA / PCA: R^2^ = 0.866; Procrustes Distance D = 0.251; P_999 permutations_ = 0.001; CVA/ bgPCA: R^2^ = 0.875; Procrustes Distance D = 0.234; P_999 permutations_ = 0.001).

#### Relationship between genetic and morphometric pattern of differentiation

Most of the specimens used for the morphometric analysis come from skull collections, without direct match with a genetic sequence. To assess the match between the morphometric and the genetic patterns of differentiation, a compromise was reached between availability of genetic data in the literature, and a geographic match between the samples used in morphometrics. Two approaches have been used. (1) Averaging group means of continental localities per country for the morphometric data set, and deleting Piana for which no genetic data were available, leading to 14 group means. (2) Duplicate genetic data obtained per country to get data for each morphometric continental locality, and assess a Corsican genetic background for Piana, presumably a sink population highly related to the nearby Corsica (18 groups, as used for morphometrics).

Both procedures led to comparable results. The CVA provided a pattern of morphometric differentiation ~1.5 closer than the one provided by the PCoA on genetic data (Table 2).

**Table 2:** Relationships between genetic and morphometric patterns of differentiation between groups. Patterns of genetic differentiation are based on coordinates of group means on the first four axes of a PCoA on the genetic distances. 14 groups: continental samples are averaged by country, Piana excluded; 18 groups: genetic data duplicated for continental samples of a same country; Piana included and considered as genetically identical to Corsica. Patterns of morphometric distances are based on the first four axes of a PCA, bgPCA and CVA on the same dataset (Fourier coefficients describing the molar shape outline). Coordinates of group means on the multivariate axes correspond to configurations compared using a Protest. It delivers a coefficient of determination (R^2^), a Procrustes distance between configurations (D_Procrustes_), and a probability P that the observed configurations are less correlated than permuted configurations (999 permutations). In italics P < 0.05, in bold P < 0.001.

Morphometrics/genetics	14 groups			18 groups		
	R^2^	D_Procrustes_	P	R^2^	D_Procrustes_	P
PCA / PCoA	0.587	0.655	*0*.*011*	0.482	0.767	*0*.*011*
bgPCA / PCoA	0.591	0.651	*0*.*009*	0.451	0.797	*0*.*042*
CVA / PCoA	0.732	0.463	**0.001**	0.677	0.542	**0.001**

### Congruence between main directions of within-group variance among well-sampled groups

The direction of main variance (Pmax) was assessed in the three well-sampled groups of Gardouch (France) and the islands Marion and Corsica. 100 bootstrapped estimates were calculated for each Pmax, providing a 95% confidence interval for the estimation of Pmax in each group. Pmax Gardouch (R_95%_ = 0.817) appeared to be less robustly estimated than Pmax in the two insular groups (R_95%_ Corsica = 0.949; R_95%_ Marion = 0.935).

The comparison between the original estimates of Pmax for these three groups showed that their correlation R fell within the 95% confidence interval of the less robustly estimated Pmax (Gardouch / Marion: R = 0.850; Gardouch / Corsica: R = 0.869; Corsica / Marion: R = 0.969). Hence, the hypothesis that Pmax are similar among groups cannot be rejected.

### Impact of similarly oriented within-group variance on PCA, bgPCA and CVA: simulations

**Fig 5:**
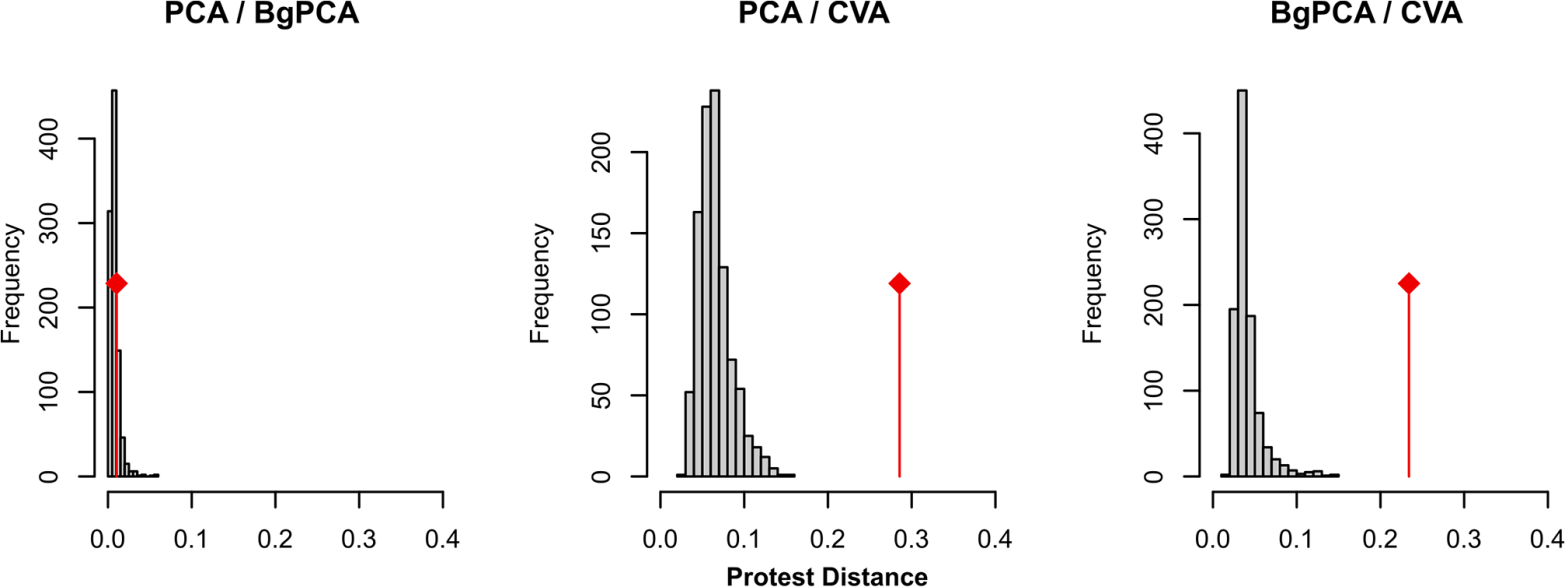
Effect of homogeneizing variances on the pattern of differentiation provided by PCA, bgPCA and CVA. The initial groups were bootstrapped, with the condition that the variance of all shape variables should follow a similar uniform distribution. The 999 resulting data sets were analysed using PCA, bgPCA and CVA. The resulting configurations on the first four axes of each analysis were compared using a Protest, providing a Procrustes distance estimating how much the configurations differ. For each pairwise comparison between analyses (PCA vs. bgPCA, PCA vs. CVA, bgPCA vs. CVA), this provided a simulated distribution (histograms in grey) for data sets with homogeneous variances. The observed distances (red lines) between analyses based on the real data set, characterized by strongly anisotropic variances for each group, were compared to these simulated distributions.

Since the CVA standardizes the within-group variance, it should be more impacted than the PCA and bgPCA by the occurrence of Pmax shared among groups. To validate this hypothesis, simulations were performed. The initial groups were bootstrapped, with the condition that the variance of all shape variables should follow a similar uniform distribution. The different groups should thus no longer share parallel Pmax. The resulting simulated configurations were compared using Protest (Fig 5). The simulated and the observed configurations provided by the PCA and the bgPCA were all very close. This shows that the PCA and bgPCA provided similar pictures of the morphometric differentiation, and that this picture was not impacted by Pmax. For the CVA cases, simulated datasets displayed configurations closer by 4 to 5 times compared to those provided by the PCA and the bgPCA than the original configuration. This is in agreement with the fact that the occurrence of a marked direction of main variance Pmax, parallel among groups, strongly impacts the pattern of differentiation as revealed by a CVA. The CVA assumes isotropic variation. In the case of anisotropic variance oriented along similar Pmax, this assumption is not fulfilled.

## Discussion

The mathematical properties of common multivariate methods such as the Principal Component Analysis and the Canonical Variate Analysis (a.k.a Linear Discriminant Analysis) are well known [15, 16]. These analyses are basic methods in the context of geometric morphometrics to visualize patterns of differentiation on which interpretative scenarios are based. The choice between methods is often rather based on the objective of expressing at best differences between the investigated groups (e.g. [6, 12, 13]).

Methodological considerations, such as the fact that the CVA standardizes the total variance by the within-group variance, are often not openly considered in this context. Yet, the CVA, by standardizing within-group variance, alters the pattern of differentiation among groups [21, 22]. This will especially occur when there is a pronounced direction of main variance Pmax parallel among groups. This does not only constitute a theoretical issue [21, 22], but indeed, Pmax more emerges as a recurrent and biologically meaningful feature in phenotypic evolution [9, 37, 61, 62]. The difference between patterns provided by PCA and CVA thus questions the (biologically meaningful) relationship between the pattern of variance in biological populations compared to differentiation between these populations.

### Within-group variance: a mathematical description, a biological feature

Multivariate axes do not correspond to any biological reality. They are mathematical ways to describe variance. However, biological interpretations rely on this description. In the case of the variance within a group, there is even a semantic and conceptual overlap between the mathematical description and the biological concept it describes. The genetic and the morphometric variances in a population are described by matrices (**G** and **P**, respectively), of which first eigenvector of each represents the direction of main variance [23, 25]. The first axis of the **G** matrix has been proposed to be a ‘line of least resistance to evolution’, because it represents the most abundant genetic variants to be screened by selection [25, 63]. Although environmental factors also act on phenotypic development, **P** seems to be a fair estimate of **G** [24, 64, 65] The first axis of the two matrices (Gmax and Pmax) appear to be correlated as well [23]. Gmax and Pmax indeed emerged repeatedly as preferred axes of evolution [23, 27, 61–63].

This means that there are biological reasons why within-group variance may not be a random noise, but be oriented along certain directions and parallel across populations [23, 62, 64]. This represents cases where the results of PCA and CVA are expected to diverge most, and it is thus likely to occur in the description of variation of biological shapes.

Questioning the involvement of Pmax in evolutionary divergence may thus be relevant to understanding the differences between results of methods applied to a same dataset. Reciprocally, contrasting the results of PCA and CVA may be enlightening to balance the role of different evolutionary processes.

### Different methods, different evolutionary patterns, all biologically relevant

**Fig 6:**
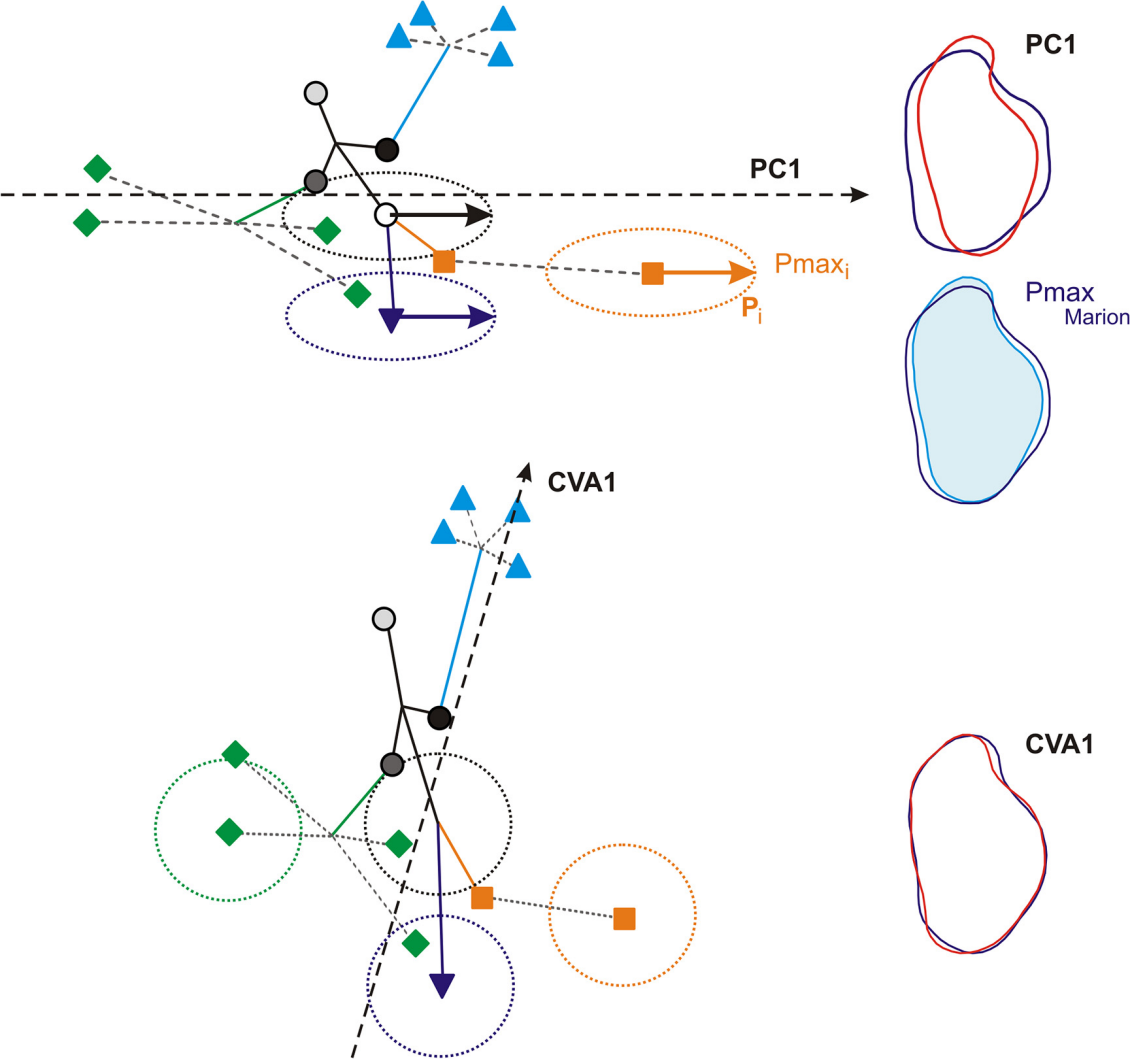
Diagrams showing how Pmax impacts the patterns of differentiation provided by a PCA and a CVA. Symbols represent group means, lines are phylogenetic relationships; full lines are ‘ancient’ branches representing differentiations of clades, dotted lines represent recent diversification in an archipelago. Dotted ellipses represent the ellipse of variance of some groups; the vector inside Pmax. Dotted vector: first axis of the multivariate analysis. Above: the first axis of a PCA is aligned with Pmax; recent diversification events occurring along this line of least resistance are highlighted. Below: the standardization of the within-group variance in a CVA compresses the differentiation along Pmax up to make the variance per group circular; this dampens the representation of diversification along Pmax and promotes patterns of phylogenetic differentiation along other axes. To the right, reconstructed outlines (using an inverse Fourier transform) exemplifying the corresponding interpretation in the cases of molar shape. Pmax corresponds to a variance from thin to broad molars; so does the first axis of the PCA. The first axis of the CVA is oblique to this direction and corresponds to more localized, detailed features on the tooth.

Considering the present case study, the PCA and the CVA highlight different evolutionary patterns in the evolution of molar shape in insular populations of house mice (Fig 4; schematic representation Fig 6). The PCA, be it on the total variance or on between-group variance, tended to promote a picture of evolution favored along lines of least resistance along the direction of main variance existing within any population (Pmax). The important divergence within the Orkney archipelago particularly exemplifies this trend, as well as the divergence of molar shape on the small islet of Piana off Corsica. In contrast, the CVA provided a pattern of evolution enhancing more basal phylogenetic divergence, such as shape features shared by all Orkney Islands. The shape changes involved (Fig 6) are a trend from slender to broad molars, when considering Pmax as divergence along the first axis of the PCA. This trend has repeatedly been shown to be a major direction of within-population variation and between-population divergence in recent and fossil murine rodents [26, 27, 62]. By standardizing by the within-group variance, the CVA puts to the front more subtle shape changes, such as a broader forepart or a backward more prominent labial cusp (Fig 6).

How general are these results? At least regarding molar shape, they may reveal some general pattern. A study of molar shape divergence in European wood mice (*Apodemus sylvaticus*) recently evidenced that insular evolution was the most prominent feature when considering total shape variation on a PCA, and that it occurred along Pmax. In contrast, phylogenetic divergence was of smaller magnitude and was not parallel to Pmax [66].

Does that point to different evolutionary mechanisms for both aspects of morphological divergence? Insular divergence is known to occur fast and to drive marked morphological divergence [67, 68]. Few founders have to face ecological conditions different from those prevailing in their source area. This presumably triggers a phase of intense selection. By providing variants available to the screening of selection, the main variance within a population constitutes a favored line of response in such a situation. Note that the morphological trend involved in Pmax may have the potential for a selective value, broader molars providing more surface to cope with abrasive matter, for instance [27]. In contrast, in most cases neutral evolution occurs at a slower pace than response to selection [69], and it should occur in any morphological direction, in an amount proportional to the frequency of the variants in the population, i.e. in any directions of variance within a population [70]. Phylogenetic divergence has no particular reason to constitute a response to selection, and may exemplify such neutral evolution, hard to detect because of slow accumulation, and in any direction, including away of Pmax. Because mice of the different clades may share similar environments while on the continent, stabilizing selection may even be involved and further dampen morphological divergence, making it difficult to perceive.

How these hypotheses may be valid for other models of evolution, i.e. for other characters and animals, are challenges for further studies. In any case, a balanced and critical confrontation of the patterns provided by the different multivariate analyses may be fruitful, when including a reflection on the biological meaning beyond morphometric variance.

## Supporting Information

S1 Table Morphometric data used in this study. Loc: Locality of trapping. UM1A1 to UM1B7: Fourier coefficients A and B of the first seven harmonic of the first upper molar (UM1) outline.
(TXT)

S1 Script Script of simulations testing the effect of homogeneizing variances on the pattern of differentiation provided by PCA, bgPCA and CVA. See results in Fig 5. The procedure is the following: The initial groups is bootstrapped, with the condition that the variance of all shape variables should follow a similar uniform distribution. The resulting data set is analysed using PCA, bgPCA and CVA. The resulting configurations on the first four axes of each analysis were compared using a Protest, providing a Procrustes distance estimating how much the configurations differ.
(R) Click here for additional data file.
